# Screening a UK amyotrophic lateral sclerosis cohort provides evidence of multiple origins of the *C9orf72* expansion^☆^

**DOI:** 10.1016/j.neurobiolaging.2014.07.037

**Published:** 2015-01

**Authors:** Pietro Fratta, James M. Polke, Jia Newcombe, Sarah Mizielinska, Tammaryn Lashley, Mark Poulter, Jon Beck, Elisavet Preza, Anny Devoy, Katie Sidle, Robin Howard, Andrea Malaspina, Richard W. Orrell, Jan Clarke, Ching-Hua Lu, Kin Mok, Toby Collins, Maryam Shoaii, Tina Nanji, Selina Wray, Gary Adamson, Alan Pittman, Alan E. Renton, Bryan J. Traynor, Mary G. Sweeney, Tamas Revesz, Henry Houlden, Simon Mead, Adrian M. Isaacs, Elizabeth M.C. Fisher

**Affiliations:** a Department of Neurodegenerative Disease, University College London, Queen Square, London, UK; b MRC Centre for Neuromuscular Diseases, University College London, Queen Square, London, UK; c Neurogenetics Unit, Queen Square, London, UK; d NeuroResource, Institute of Neurology, University College London, Queen Square, London, UK; e Queen Square Brain Bank for Neurological Disorders, University College London, Queen Square, London, UK; f MRC Prion Unit, University College London, Queen Square, London, UK; g Department of Molecular Neuroscience, University College London, Queen Square, London, UK; h National Hospital for Neurology and Neurosurgery, Queen Square, London, UK; i Centre for Neuroscience & Trauma, Blizard Institute, Barts and The London School of Medicine and Dentistry, Queen Mary University of London, UK; j Sobell Department of Motor Neuroscience and Movement Disorders, University College London, Queen Square, London, UK; k Neuromuscular Diseases Research Section, Laboratory of Neurogenetics, National Institutes of Health, National Institute on Aging, Bethesda, MD, USA

**Keywords:** Frontotemporal dementia, Somatic instability, Amyotrophic lateral sclerosis

## Abstract

An expanded hexanucleotide repeat in the *C9orf72* gene is the most common genetic cause of amyotrophic lateral sclerosis and frontotemporal dementia (C9ALS/FTD). Although 0–30 hexanucleotide repeats are present in the general population, expansions >500 repeats are associated with C9ALS/FTD. Large C9ALS/FTD expansions share a common haplotype and whether these expansions derive from a single founder or occur more frequently on a predisposing haplotype is yet to be determined and is relevant to disease pathomechanisms. Furthermore, although cases carrying 50–200 repeats have been described, their role and the pathogenic threshold of the expansions remain to be identified and carry importance for diagnostics and genetic counseling. We present clinical and genetic data from a UK ALS cohort and report the detailed molecular study of an atypical somatically unstable expansion of 90 repeats. Our results across different tissues provide evidence for the pathogenicity of this repeat number by showing they can somatically expand in the central nervous system to the well characterized pathogenic range. Our results support the occurrence of multiple expansion events for C9ALS/FTD.

## 1 Introduction

Expansions of a GGGGCC hexanucleotide repeat in the first intron and/or promoter region of *C9orf72* are the most frequent known monogenic cause of amyotrophic lateral sclerosis (ALS) and frontotemporal dementia (FTD) in populations of European descent (DeJesus-Hernandez et al., 2011, Gijselinck et al., 2012, Majounie et al., 2012, Renton et al., 2011). Repeats ranging in size from 0 to 30 are found in the general population, whereas pathogenic expansions range between 500 and 4500 repeats, with considerable somatic instability (Beck et al., 2013, DeJesus-Hernandez et al., 2011, Van Blitterswijk et al., 2013b). The finding of a conserved haplotype spanning approximately 200 kb around pathogenic expansions may result from either a common founder, or a “risk” haplotype predisposing to expansion (Beck et al., 2013, Majounie et al., 2012, Mok et al., 2012, Pliner et al., 2014). How the *C9orf72* expansion leads to disease is as yet unknown, proposed mechanisms include the following: (1) loss of *C9orf72* function; (2) RNA toxicity associated with the presence of nuclear repeat expansion RNA foci; and (3) protein toxicity caused by dipeptide repeats originating from the translation of the repeat expansion (Ling et al., 2013).

We present genetic and clinical data from *C9orf72* screening in a UK ALS cohort. We confirm the clinical features previously associated with *C9orf72* expansions and report an atypical expansion with approximately 90 repeats in blood which shows somatic instability within and between tissues. Our analysis of multiple tissues shows the repeat significantly expanded in the central nervous system (CNS) to over 3000 hexanucleotides.

Our data, together with recently reported cases, strongly support the hypothesis that *C9orf72* repeat expansions occur in different individuals in the context of the permissive risk haplotype (Dols-Icardo et al., 2014, Van Blitterswijk et al., 2013b). This finding sheds light on the origins of the *C9orf72* repeat expansion and has important implications for pathomechanisms of *C9orf72* ALS/FTD, diagnostic testing, and genetic counseling for *C9orf72* expansions.

## 2 Methods

We screened a cohort of 452 ALS patients who had not been screened for other genetic causes of ALS, from University College London Partners Motor Neuron Disease clinics (London, UK). Patients gave written consent, and the project was approved by the local ethical review committee. DNA was extracted from blood and flash-frozen tissues using standard techniques.

Fibroblasts were generated from a 3-mm skin punch biopsy taken under local anesthetic following informed consent. Biopsies were dissected into 1 mm pieces and cultured at 37 °C, 5% CO_2_ in DMEM, 10% FBS, 1% L-Glutamine, 50U/mL penicillin, and 50 μg/mL streptomycin until fibroblasts were seen to grow out from the explants. Media changes were performed every 3 days. When fibroblasts reached confluency, they were detached from culture dishes using TrypleE (Invitrogen) and transferred to larger culture vessels for further expansion. Cells (1 × 10^7^) were used for genomic DNA extraction using the standard techniques.

**Fig. 1:**
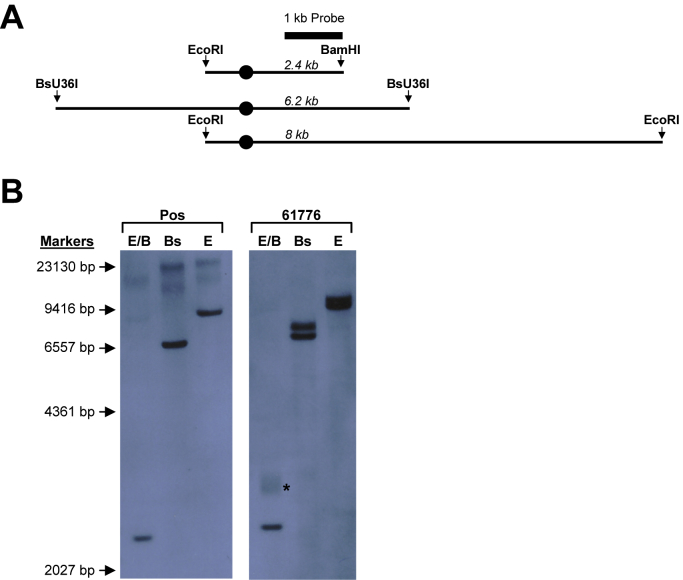
Case 61776 shows a somatically unstable repeat. (A) Map of the *C9orf72* locus illustrating the probe and the restriction sites used for single-copy 1-kb probe Southern hybridization. The site of the hexanucleotide expansion is indicated with a circle. (B) Southern hybridization performed on a typical bimodal *C9orf72* repeat expansion (Pos) and case 61776 with the following restriction digestions: EcoRI/BamHI (E/B), Bsu36I (Bs), and EcoRI (E). The somatic mosaicism in 61776 is evident in the E/B digest lane (marked with *).

Repeat-primed polymerase chain reaction expansion screening and single-nucleotide polymorphism (SNP) genotyping was performed on blood-derived DNA as previously described (Beck et al., 2013, Majounie et al., 2012). Genetic screening data for 350 patients were previously reported (Beck et al., 2013). Two Southern hybridization methods for expansion sizing were performed using: (1) an oligonucleotide probe consisting of 5 GGGGCC repeats (Beck et al., 2013); or (2) a single-copy 1 kb probe (Fratta et al., 2013) that anneals adjacent to the repeat (see Supplementary Table 1 for PCR-primer sequences used to derive the single-copy probe). Three different restriction enzyme digestions were used in the single-copy probe method to produce “normal” bands of different sizes: EcoRI/BamHI double digest: 2.4 kb; BsU36I: 6.2 kb; EcoRI: 8 kb (Fig. 1A); the 6.2 kb and 8 kb bands compress the large somatic smears in expansion-positives for unambiguous detection, whereas the 2.4-kb band allows accurate sizing.

Case 61776 was donated to the NeuroResource tissue bank, UCL Institute of Neurology. Routine neuropathologic assessment was carried out in the Queen Square Brain Bank for Neurological Disorders (Lashley et al., 2011). Formalin-fixed and paraffin-embedded tissue sections (7 microns thick) were immunostained as previously described using commercially available antibodies to the following proteins: TDP-43 (Abnova, Taipei City, Taiwan; 1:800), p62 (BD Transduction Laboratories, Oxford, UK; 1:200), ubiquitin (Dako, Ely, UK; 1:200), α-synuclein (Vector, Peterborough, UK; 1:50), Aβ (Dako; 1:100), tau (AT8 clone; Autogen Bioclear, Wiltshire, UK; 1:600), CD68 (Dako; 1:150), and GFAP (Dako; 1:1000) as previously described (Lashley et al., 2011). Sections were also immunostained with novel antibodies able to detect each of the proteins generated by non-ATG dependent translation of the noncoding repeat expansion. Antibodies, made in rabbits by Biogenes (Germany), were generated against (Gly-Ala)_7_, (Gly-Pro)_7_, (Gly-Arg)_7_, (Pro-Arg)_7_, or (Pro-Ala)_7_ peptides.

Fluorescence in situ hybridization was performed with 2′-O-methyl RNA probes (Integrated DNA Technologies): (GGCCCC)4 for sense and (GGGGCC)4 for antisense RNA foci, 5′ labeled with Cy3 or Alexa488, respectively. In parallel with staining with NeuN (ABN78, Millipore; 1:250) to identify neurons was performed as previously described (Mizielinska et al., 2013).

## 3 Results

### 3.1 Clinical features and *C9orf72* screening of a UK ALS cohort

We screened *C9orf72* hexanucleotide repeats in 452 ALS patient blood-derived DNA samples using repeat-primed polymerase chain reaction and revealed expansions of >30 repeats in 30 patients (6.6%), hereafter referred to as C9+ individuals. The mean age at onset of C9+ cases was 54.6 years compared with 59.3 years in noncarriers. Site of disease onset was bulbar in 26% of C9+ cases compared with 27% in noncarriers (Table 1).

**Table 1:** Summary of clinical features of the general cohort and *C9orf72* expansion positive individuals

	*C9orf72* expansion negative patients^a^ (n = 422)	*C9orf72* expansion positive patients^b^ (n = 30)
Male:female	1.55	2.00
Age at onset ± SD (y)	59.3 ± 12.5	54.6 ± 8.1
Bulbar onset (%)	27.3	26.1
Positive family history (%)	3.8	30.0

Key: SD, standard deviation.

a Site of onset and age of onset missing for 81 patients and gender missing for 62 patients.

b Site of onset and gender missing for 7 patients and gender missing for 3 patients.

### 3.2 Sizing of C9orf72 repeat reveals one exception to very large expansions

Southern hybridizations were performed on blood DNA from 27 C9+ cases (19 previously reported) (Beck et al., 2013; see Supplementary Fig. 1 for details on the additional 8) and confirmed the presence of somatically unstable expanded repeats with sizes between 1100 and 4200. One exception was case 61776 which showed a doublet indicating a small expansion of the *C9orf72* repeat (Beck et al., 2013). To better discriminate the size of the expanded repeat, we performed single-copy probe Southern blotting and established that this individual carried an expansion of approximately 90 repeats in blood. The EcoRI/BamHI double digest, electrophoresed as a smear indicating somatic instability (70–120 repeats) (Fig. 1B).

### 3.3 Case 61776 shows typical C9orf72 molecular and histopathologic features in postmortem brain

The clinical features of case 61776, outlined in Supplementary Material were typical of ALS, with no family history of the disease, bulbar onset at 57 years, and a 6-year disease course before death.

**Fig. 2:**
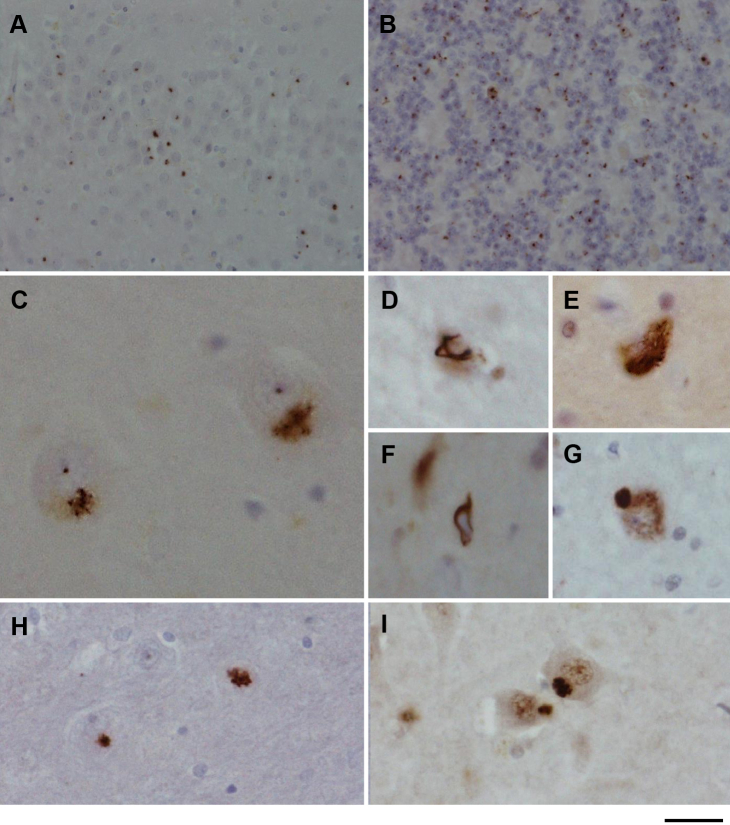
Immunohistochemical analysis of case 61776. Small p62-positive “star-like” inclusions were present in the granule cells of the dentate fascia (A) and the cerebellar cortex (B). Larger p62-positive inclusions were seen in the CA4 subregion of the hippocampus and also contained intranuclear inclusions (C). TDP-43-positive oligodendroglial inclusions were seen in the striatum (D) and substania nigra (F). TDP-43-positive neuronal cytoplasmic inclusions were present in the 12th nerve nucleus (E) and spinal cord (G). The “star-like” p62-positive inclusions were also positive with anti-glycine proline (H) and anti-glycine arginine dipeptide antibodies (I). Bar represents 50 μm in A and B; 20 μm in E, G, H, and I; and 10 μm in C, D, and F.

Postmortem neuropathologic analysis revealed mild frontal atrophy and a discoloration of the anterior horn in the spinal cord. Numerous p62-positive and TDP-43 negative star-like neuronal cytoplasmic inclusions were seen in the granule cells of the dentate fascia, hippocampal subregions, and cerebellar cortex (Fig. 2A and B). These were shown to contain the dipeptide proteins (Fig. 2H and I). An occasional TDP-43-positive inclusion was seen in oligodendrocytes of the pencil fibers of the striatum and substantia nigra (Fig. 2D and F). The neurons of the XII cranial nerve nucleus were depleted with a single TDP-43 neuronal cytoplasmic inclusion seen (Fig. 2E and G). Thoracic spinal cord showed severe depletion of the anterior horn neurons together with degeneration of both the crossed and uncrossed corticospinal tracts. Occasional TDP-43-positive cytoplasmic inclusions were observed in surviving motor neurons along with fine neuropil threads and occasional oligodendroglial cells with fine filamentous intracytoplasmic inclusions.

**Fig. 3:**
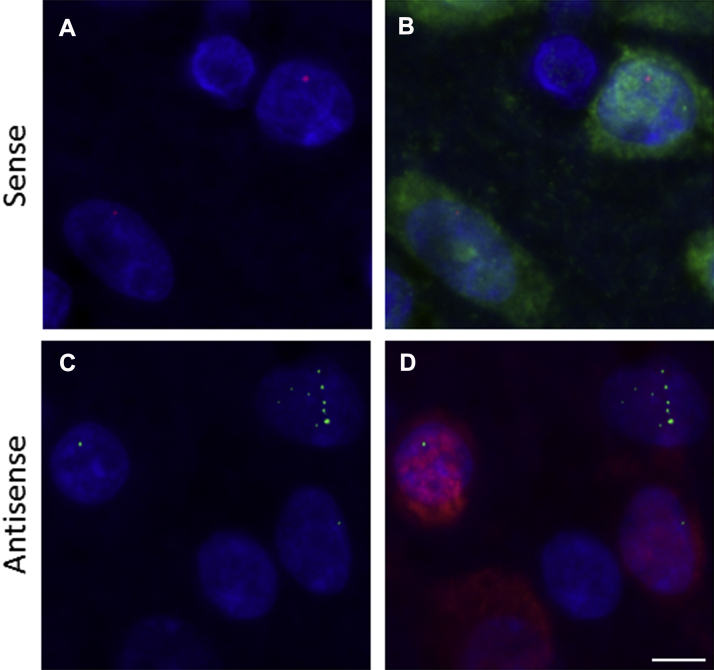
Fluorescence in situ hybridization of case 61776. RNA foci containing sense (A and B) and antisense (C and D) C9orf72 expansion transcripts are present in the frontal cortex. Neurons are identified by NeuN staining in green (B) and in red (D). Scale bar = 5 μm.

RNA foci containing sense and antisense *C9orf72* expansion transcripts are a characteristic feature of *C9orf72* ALS/FTD. To investigate the presence of RNA foci in this case, fluorescence in situ hybridization was performed. We detected abundant sense and antisense foci in the frontal cortex (Fig. 3) and also in the hippocampus, cerebellum, and spinal cord (data not shown).

### 3.4 An expansion of the C9orf72 hexanucleotide repeat has occurred in the CNS

**Fig. 4:**
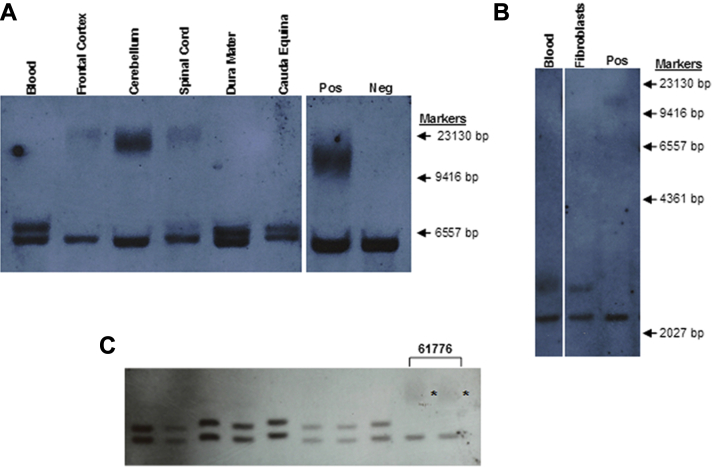
Single-copy probe Southern blotting of case 61776. (A) Southern blots of DNA from various tissues, DNA digested with BsU36I. The expansion to a large repeat has occurred in the frontal cortex, cerebellum, and spinal cord. (B) Blood and fibroblast DNA double-digested with BamHI/EcoRI. The fibroblasts only possess the 90 repeat allele and not the large expansion. (C) Southern blots following BamHI/EcoRI digestions performed on individuals with repeat sizes of, in order on the blot, 2+20, 2+22, 2+24, 5+26, 10+25, 2+23, 2+23, and 5+27 repeats (sized by PCR), and 2 independent blood DNA extractions from case 61776 (mosaic band marked with *) whose nonexpanded allele has 5 repeats. No mosaicism was shown in the cases with 20–27 repeats. The position of the bands did not reflect their size relative to each other in all cases: the electrophoresis of the bands where more DNA was loaded was retarded in the agarose gel. Abbreviations: Neg, negative control; PCR, polymerase chain reaction; Pos, positive control.

We performed Southern blots on DNA derived from postmortem flash-frozen CNS samples from case 61,776. Frontal cortex, cerebellum, and spinal cord-derived DNA showed very large *C9orf72* repeat expansions (approximately 950–3000+ repeats), not differing from typical *C9orf72* cases, with the exception of the presence in the spinal cord DNA of a band representing the 90 repeat expansion together with the larger repeat (Fig. 4A).

In the absence of parental DNA, to answer whether the hexanucleotide repeat had expanded specifically in the CNS, or whether there had been a retraction in a regenerating tissue such as blood, where a selective pressure can be envisaged, we also analyzed the *C9orf72* repeat size in tissues and cell types with diverse embryological derivations, including dura mater (mesoderm or a proposed dual mesoderm-ectoderm origin) (Adeeb et al., 2012, Kaplan et al., 2005), cauda equina (ectoderm), and skin-derived fibroblasts (mesoderm) (Fig. 4A and B). All samples showed the presence of the smaller 90 repeat expansion similar to that in blood (mesoderm). These results suggest the repeat has expanded in size in the developmental lineage after the differentiation between central and peripheral nervous system and making the possibility of a retraction to the same size in multiple tissues less likely.

### 3.5 Assessing the threshold for somatic repeat expansion instability

Previous reports have indicated that 20–30 hexanucleotide repeat expansions, commonly referred to as “intermediate” repeats, play a pathogenic role (Gómez-Tortosa et al., 2013). To assess if similar somatic instability occurs in these repeats, we performed Southern blotting on 8 samples with 20–27 repeat expansions and showed no detectable instability in this size range (Fig. 4C).

### 3.6 A permissive haplotype and multiple expansion events

We then assessed if case 61776 carried the risk haplotype found to associate with all known cases of *C9orf72* ALS/FTD and performed genetic analysis of 36 of the 42 SNPs that define this “Finnish risk” haplotype (Majounie et al., 2012). Results showed that case 61776 carried the risk allele on a consecutive stretch of 22 SNPs spanning 105 kb upstream to 26 kb downstream of the *C9orf72* gene (Supplementary Table 2), therefore not differing from other *C9orf72* positive cases.

### 3.7 Atypical C9orf72 repeat expansions occur at low frequency in blood

To estimate the frequency of atypical repeat expansions, we reviewed all *C9orf72* Southern blotting data published until the beginning of March 2014. Of 345 C9+ ALS/FTD probands (where expansion size was investigated by Southern blotting), 13 showed atypical expansions of 50–200 repeats (3.8%), including 2 cases where the 50–200 repeat allele occurred in conjunction with a large expansion (Buchman et al., 2013, Waite et al., 2014). A number of these blots were performed on DNA derived from lymphoblastoid cell lines which are known to poorly represent the range of repeats in vivo (Beck et al., 2013, Hübers et al., 2014). If only Southern blots performed on blood DNA are considered, 6/195 expansions fall within the 50–200 repeat range (3.1%) (Supplementary Table 3).

## 4 Discussion

We present clinical features and *C9orf72* expansion frequency and sizing data for a cohort of 452 UK ALS patients. In accordance with previous reports, the age at onset in the C9+ group is younger than C9− cases (Majounie et al., 2012). In our cohort, bulbar onset prevalence was not increased in C9+ cases. The frequency of *C9orf72* repeat expansions has been found to vary between different geographical regions (Pliner et al., 2014, Woollacott and Mead, 2014); in our cohort, the frequency (∼7%) is similar to that reported in previous UK studies (Cooper-Knock et al., 2012).

Although the presence of *C9orf72* repeat expansion has been tested in numerous studies, only a minority have used Southern blotting to size the hexanucleotide expansion (Beck et al., 2013, Buchman et al., 2013, DeJesus-Hernandez et al., 2011, Dobson-Stone et al., 2013, Dols-Icardo et al., 2014, Fratta et al., 2013, Harms et al., 2013, Hensman Moss et al., 2014, Hübers et al., 2014, p. 72; Ishiura et al., 2012, Mann et al., 2013, Meisler et al., 2013, Murray et al., 2013, Takada et al., 2012, Van Blitterswijk et al., 2013a, Van Blitterswijk et al., 2013b, Waite et al., 2014). Overall, these have shown that in DNA extracted from the most commonly analyzed tissues, such as blood, cerebellum, and frontal cortex, most of the expansions range between several hundred to several thousand repeats. The largest smears were typically observed in blood, and a larger, tighter band commonly observed in frontal cortex, compared with a smaller, more diffuse smear in the cerebellum (Beck et al., 2013, Van Blitterswijk et al., 2013b). In rare cases, expansions of approximately 50–200 repeats have been reported in blood DNA (Buchman et al., 2013, Dobson-Stone et al., 2013, Dols-Icardo et al., 2014, Van Blitterswijk et al., 2013b). The pathogenicity of such size repeats and the corresponding repeat size in CNS tissues in some of these cases remains to be determined.

Our analysis of the *C9orf72* repeat expansion size in blood confirms the presence of several hundred to several thousand repeats in most of the cases, but we also describe a single case carrying approximately 90 repeats in blood and other tissues. The postmortem analysis on this case shows the typical *C9orf72* pathology pattern and interestingly, Southern blotting of frontal cortex, cerebellum, and spinal cord regions showed an expansion much larger than in blood and in the typical range of *C9orf72* cases.

Our Southern blot analysis performed on cells and tissues from diverse embryological origins strongly suggests that an expansion from 90 repeats to 950–3000+ repeats occurred in the CNS. Indeed, the large expanded repeat is present only in brain and spinal cord, whereas the 90 repeat expansion is present both in ectodermally derived cauda equina and in more “distantly-related” tissues such as fibroblasts, blood and dura mater that originate from the mesoderm and mesoderm and/or ectoderm. These findings are consistent with those recently published by Van Blitterswijk et al. (2013b) who presented a case (P20) with a small expansion in several non-CNS tissues and large expansions in CNS tissue.

In both cases, the “small” expanded alleles are very similar in size among tissues, making the possibility of a retraction of the very large expansion to one of approximately 90 repeats unlikely. Indeed our findings demonstrate this would involve multiple retraction events (e.g., in the mesoderm and ectodermal peripheral nervous system progenitors) and, further, all retraction events would need to lead to the same final size.

Dols-Icardo et al. (2014) have described a family where 3 brothers carry repeats ranging from 116 to 148 in blood, with offspring of 2 of the brothers inheriting repeats of 120 and 1401 repeats. This finding brings further support to the “multiple origin” hypothesis of *C9orf72* large expansions.

The presence of the typical Finnish haplotype in the case here described supports the possibility that a permissive allele, predisposes to the hexanucleotide repeat expansion.

Another possible explanation, compatible with the single founder hypothesis, is that the original expansion occurred to a size of approximately 90 repeats, which expanded further in multiple cases. Given the instability of such repeats within the individual described here, and between generations (Dols-Icardo et al., 2014), this appears unlikely.

Whether retractions may also occur in *C9orf72* cases remains a possibility. Van Blitterswijk et al. (2013b) have reported 2 cases in which multiple tissues have large expansions with the exception of a smaller band in either skeletal muscle (P14) or testes (P25). In these cases, single retraction events in those tissues may be possible.

The pathogenic threshold of the *C9orf72* expansion remains to be determined. The findings reported here underline the necessity to study both peripheral and CNS tissues to identify this. Expansions ranging from 20 to 22 repeats have been associated with FTD (Gómez-Tortosa et al., 2013), but further segregation data and screening of large numbers of patients and controls are needed to clarify their significance. Whether these repeats expand somatically also remains to be addressed, but it is important to note that alleles of 20–27 repeats, in contrast to case 61776, did not show somatic instability in our study. The presence of somatic instability in blood does not offer direct evidence of pathogenicity, but is an important feature to consider, because in many neurologic disorders caused by nucleotide repeat expansions, pathogenic expansions are characterized by somatic instability, often with greater instability in tissues directly related to disease (Brouwer et al., 2009, Kennedy et al., 2003).

In diagnostic testing for Huntington disease, spinocerebellar ataxias types 1, 2, 3, 7, Friedreich ataxia, dentatorubral-pallidoluysian atrophy, and X-linked spinal bulbar muscular atrophy, our laboratory has screened over 40,000 alleles and observed somatic instability in DNA extracted from blood in all pathogenic repeats but none from alleles with repeats in the nonpathogenic range. Taken together, these data suggest the threshold for instability may lie between >30 and 90 repeats.

Our review of all published C9+ cases underlines the fact that atypical expansions detected in blood represent approximately 3.1% of cases. Genetic counseling for these patients is currently challenging, because CNS tissues have not been examined in all published cases and the pathogenic repeat threshold and the likelihood of expansion in the brain specifically is unknown. Future studies addressing further aspects of the hexanucleotide expansions, such as the methylation state of the repeat itself and surrounding regions, may also contribute to the discrimination between pathogenic and nonpathogenic expansions (Xi et al., 2013).

We provide additional evidence that the *C9orf72* expansion has occurred on multiple occasions on a permissive haplotype. That somatic instability may play a fundamental role in certain cases highlights the importance the understanding of this mechanism may have and may point to possible therapeutic strategies.
