# Supplementary material for: Screening a UK amyotrophic lateral sclerosis cohort provides evidence of multiple origins of the C9orf72 expansion
Source: Neurobiol Aging. 2015 Jan;36(1):546.e1–7. doi: 10.1016/j.neurobiolaging.2014.07.037 (PMC4270445; doi:10.1016/j.neurobiolaging.2014.07.037)
Supplement: Supplementary Material [file mmc1.pdf]

Supplementary Table 1: PCR oligonucleotide primer sequences used to generate the 1 kb single-copy Sothern blot probe.

Primer Name	Sequence
C9orf72_1kbProbe_F	AACCGTCCACTTTCCACAAC
C9orf72_1kbProbe_R	TCACAGCTCCGAGATGACAC

Supplementary Table 2 – Finnish risk haplotype in case 61776. Shading highlights the shared haplotype region (continued overleaf).

dbSNP	Chr.	Coord.	Ref	Alt	Fin_haplotype	Case 61776
rs1330921	9	27367278	T	C	T	CT
rs10121765	9	27372053	A	C	C	CC
rs1110264	9	27385002	G	A	A	GG
rs1110155	9	27386505	A	C	A	AA
rs2150336	9	27402961	T	C	C	TT
rs2225389	9	27409264	A	G	G	AA
rs1161680	9	27430232	G	A	G	AA
rs2120718	9	27438411	G	A	G	GG
rs1058326	9	27447089	C	T	C	TT
rs765709	9	27451960	A	C	A	AA
rs1316679	9	27452240	G	A	G	GG
rs4406503	9	27453037	G	A	G	GG
rs10511817	9	27454173	C	A	C	CC
rs725804	9	27458939	C	A	A	CC
rs10511816	9	27468461	G	T	T	TG
rs1444533	9	27477874	G	A	A	AA

dbSNP	Chr.	Coord.	Ref	Alt	Fin_haplotype	Case 61776
rs1822723	9	27478052	C	T	C	CC
rs4879515	9	27482235	C	T	T	TT
rs895023	9	27483959	C	T	T	TT
rs7046653	9	27490967	A	G	A	AG
rs2440622	9	27495418	C	A	A	AA
rs1977661	9	27502986	C	A	C	CC
rs903603	9	27529316	C	T	C	CC
rs10812610	9	27533984	C	A	C	CC
rs2814707	9	27536397	G	A	A	AG
rs3849942	9	27543281	A	G	A	AG
rs12349820	9	27553876	T	C	T	TT
rs1565948	9	27559733	G	A	G	AG
rs774359	9	27561049	T	C	C	CT
rs2282241	9	27572255	G	T	G	TG
**C9ORF72_expansion**	**9**	**27573527-27573544**	**3**	**>30**	**>30**	**~90**
rs1948522	9	27575785	C	T	C	TC
rs1982915	9	27579560	A	G	G	GG
rs2453556	9	27586162	A	G	G	GG
rs702231	9	27588731	C	A	A	CA
rs696826	9	27589657	A	G	G	AG
rs2477518	9	27599746	T	C	T	CT

Supplementary Table S3: All published *C9orf72* expansion-positive cases Southern blotted up to March 2014, including all alleles reported in the range 50-200 GGGGCC repeats. Only index cases from each study are included and cases reported in more than one publication have only been included once. The number of blood samples from each study and the corresponding number of GGGGCC repeats in the range 50-200 is also shown. B = Blood, BR = Brain (unspecified), BS = Brain Stem, CB = Cerebellum, CC = Cerebral Cortex, CE = Cauda Equina, DM = Dura Mater, FB = Fibroblasts, FC = Frontal Cortex, H = Heart, L = Liver, LB = Lymphoblastoid Cell Line, M = Muscle, OC = Occipital Cortex, S = Spleen, SC = Spinal Cord, T = Testes, TC = Temporal Cortex, U = Unknown. * = Report the same case, which has only been counted once in the 'Total' row.

Citation	Total index cases	Total expansions 50-200 rpts	Blood samples	Blood samples with expansions 50-200 rpts	All tissues/cell lines blotted	Tissues/cell lines 50-200 rpt alleles detected in	Approx. repeat size of 50-200 rpt alleles
This report	9*	1*	9*	1*	B,CB,CE,DM,FC,FB,SC	B,CE,DM,SC	90
Hubers et al 2014	34	-	17	-	B,LB	-	-
Waite et al 2014	15	2	2	-	B,CB,FC,LB	1:CB; 2:LB	1: 130-300; 2: 60
Dols-Icardo et al 2014	60	3	56	3	B,CB	B	1:148; 2:130; 3:192
Hensman Moss et al 2014	2	-	2	-	B	-	-
Mann et al 2013	12	-	-	-	BR	-	-
van Blitterswijk 2013a	1	-	-	-	CB,FC,TC	-	-
van Blitterswijk 2013b	84	3	47	1	B,CB,FC,H,L,OC,M,S,SC,T	1:S,H,M,B,L,SC; 2:M; 3:T	Not Sized
Murray et al 2013	1	-	-	-	FC,TC	-	-
Fratta et al 2013	1	-	-	-	B,BR	-	-
Meisler et al 2013	1	-	1	-	B,LB	-	-
Harms et al 2013	8	-	-	-	FB,OC	-	-
Buchman et al 2013	34	3	5	-	B,CB,CC,LB	LB	50
Beck et al 2013	69*	1*	48*	1*	B,BR,BS,CB,FC,LB,U	B	90
Dobson-Stone et al 2013	6	1	6	1	B	B	65
Ishiura et al 2012	3	-	-	-	LB	-	-
Takada 2012	1	-	1	-	B	-	-
DeJesus-Hernandez et al 2011	5	-	2	-	B,BR,LB	-	-
**Total**	**345**	**13 (3.8%)**	**195**	**6 (3.1%)**			

Supplementary text: ***Clinical details of Case 61776, patient carrying the C9orf72 90 repeat expansion***.

Patient presented at 57 years of age with progressive speech difficulties and occasional choking episodes. 1.5 years after onset, developed widespread fasciculations of the tongue and upper limbs and diffuse brisk reflexes. EMG was compatible with anterior horn disease and the diagnosis of motor neuron disease was made. Following year progressed to develop a head drop and proximal and distal wasting of the upper limbs with a “split hand” pattern. 2.5 years after onset noticed initial difficulty in climbing stairs and lower limb weakness. Started using a wheelchair 3 years after onset and became wheelchair dependant six months later. 4.5 years after onset underwent PEG surgery and started using NIV. Reported autonomic dysfunction. Died 6 years after onset.

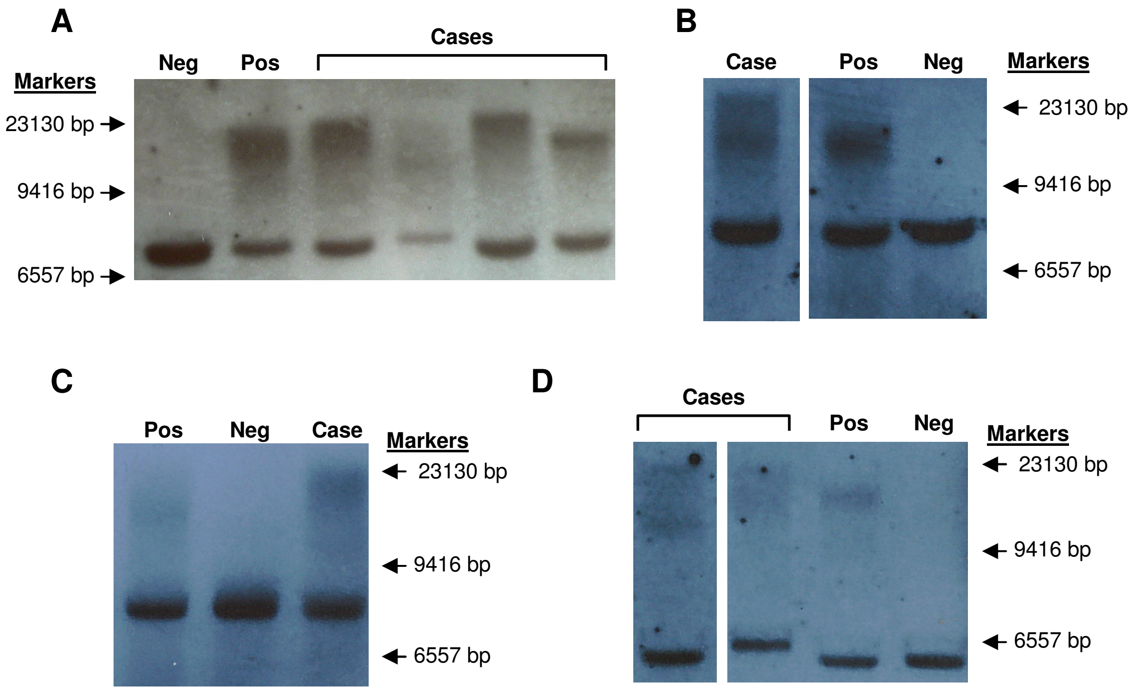

Supplementary Figure 1 – Southern blots of blood DNA samples from the eight *C9orf72* expansion-positive cases not reported in Beck et al 2013, with negative (Neg) and positive (Pos) controls. DNA digestion was performed with EcoRI (A,B and C) or BsU36I (D), probing with the 1-kb single-copy probe (see Fig 1A). The mobility of the digested DNA in the 3rd case in image D is different to the other DNAs, with the normal band noticeably higher. This DNA sample was from frozen blood that had been extracted by a different method compared to the other samples. Sizing and RP-PCR confirmed that the normal allele has 2 repeats (data not shown).
